# The availability of purine nucleotides regulates natural competence by controlling translation of the competence activator Sxy

**DOI:** 10.1111/mmi.12245

**Published:** 2013-05-13

**Authors:** Sunita Sinha, Joshua Mell, Rosemary Redfield

**Affiliations:** Department of Zoology, University of British ColumbiaVancouver, V6T 3Z4, Canada

## Abstract

Many bacteria are naturally competent, able to bind and take up DNA from their extracellular environment. This DNA can serve as a significant source of nutrients, in addition to providing genetic material for recombination. The regulation of competence in several model organisms highlights the importance of this nutritional function, although it has often been overlooked. Natural competence is induced by starvation in *Haemophilus influenzae*, the model for competence regulation in the gamma-proteobacteria. This induction depends on the activation of the global metabolic regulator CRP, which occurs upon depletion of phosphotransferase sugars. In this work, we show that the depletion of purine nucleotides under competence-inducing conditions activates the CRP-dependent competence-specific regulator Sxy. Depletion of extra- or intra-cellular purine nucleotides activates Sxy translation, while high levels inhibit it. This is modulated by the stem structure formed by *sxy* mRNA. The exact mechanism by which the nucleotide depletion signal is transduced is unclear, but it does not involve direct binding of purine intermediates to the *sxy* stem, and does not require Hfq or competence proteins. Similar regulation occurs in the relatives of *H. influenzae, Actinobacillus pneumoniae* and *A. suis,* confirming the importance of processes enabling competent bacteria to exploit the abundant DNA in their environments.

## Introduction

Natural competence is the ability of bacteria to actively take up DNA from their environment. Since this DNA can recombine with the chromosome and change the cell's genotype (transformation), natural transformation is a major mechanism of genetic exchange, shaping bacterial genomes and spreading alleles that increase bacterial survival and virulence (Domingues *et al*., 2012; Livermore, 2012). However, most DNA taken up by competent cells is degraded, providing nucleotides, elements (C, N, P) and energy, but no genetic information (Pifer and Smith, 1985; Stewart and Carlson, 1986). This suggests that DNA uptake could make a significant contribution to cellular metabolism. However, although nutritional signals affect competence development in most model systems, their roles are often thought to be indirect (Solomon and Grossman, 1996; Macfadyen, 2000; Finkel and Kolter, 2001; Palchevskiy and Finkel, 2006; Bosse *et al*., 2009; Johnsborg and Havarstein, 2009; Kristensen *et al*., 2012).

Nutritional signals play a prominent role in competence of *Haemophilus influenzae*, the model for gamma-proteobacteria. The traditional method for competence induction abruptly deprives cells of both nucleotides and the carbon/energy resources needed for nucleotide synthesis (Maughan *et al*., 2008). On the molecular level, when phosphotransferase (PTS) sugars are unavailable, a rise in intracellular cAMP levels activates CRP (cAMP Receptor Protein) to induce transcription of sugar utilization genes (containing the canonical CRP-N binding site) and of the competence activator *sxy* (Dorocicz *et al*., 1993; Macfadyen, 2000; Redfield *et al*., 2005). Sxy and CRP together then activate transcription of the 25 genes in the CRP-S regulon (containing the non-canonical CRP-S binding site), whose products enable the cell to take up DNA (Redfield *et al*., 2005; Cameron and Redfield, 2008; Sinha *et al*., 2012).

Though the PTS sugar depletion cascade that controls cAMP and CRP is well understood, the signals and mechanisms that control Sxy translation are less clear. In addition to transcriptional control by CRP, Sxy is subject to translational control by an mRNA stem structure (Cameron *et al*., 2008). Competence development requires the unfolding of this stem, and mutants in which the stem is weakened are hypercompetent (Cameron *et al*., 2008). In *Vibrio cholerae* and its relatives, where the regulation of competence and of Sxy has also been extensively studied, a similar stem in the *sxy* homologue *tfoX* (Yamamoto *et al*., 2010) transduces a nutritional signal, the availability of the N-acetylglucosamine polymer chitin (Meibom *et al*., 2005; Pollack-Berti *et al*., 2010; Yamamoto *et al*., 2010; 2011; Antonova and Hammer, 2011; Suckow *et al*., 2011; Antonova *et al*., 2012; Blokesch, 2012; Lo Scrudato and Blokesch, 2012; Seitz and Blokesch, 2012). In response to chitin, a small Hfq-regulated RNA, *tfoR*, modulates *tfoX* translation by base pairing with part of the stem (Yamamoto *et al*., 2011).

Although the regulatory inputs controlling *H. influenzae sxy* translation are not known, depleted nucleotide pools have been implicated in competence induction. The starvation medium that maximally induces competence (MIV) lacks nucleotides, and microarray studies have confirmed that the abrupt transfer from rich medium to MIV sharply induces purine biosynthesis genes (Herriott *et al*., 1970; Redfield *et al*., 2005). In addition, Macfadyen *et al*. showed that the addition of extracellular purine nucleotides represses natural competence (Macfadyen *et al*., 2001). The biggest repressing effect was seen with the nucleotides AMP and GMP and the nucleoside guanosine, while adenosine had a smaller effect and bases had little; this difference was attributed to differences in the transport process (Macfadyen *et al*., 2001).

**Fig. 1:**
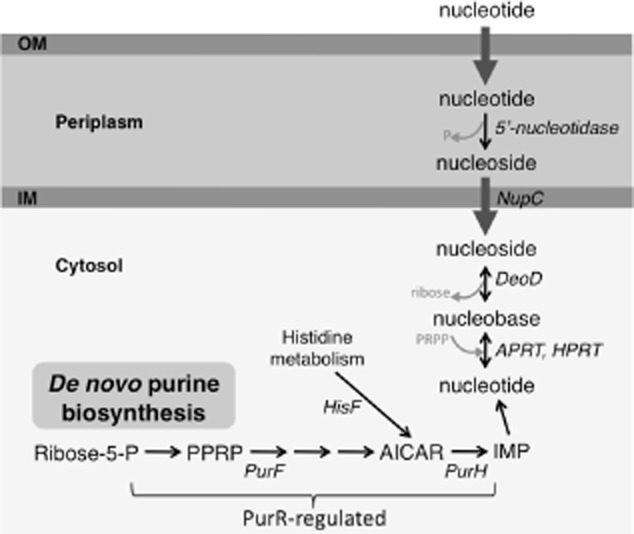
Purine nucleotide transport and metabolism in *H. influenzae*. Both extracellular purine pools and endogeneous purine biosynthesis affect purine nucleotide levels in the cytoplasm. Extracellular nucleotides are transported across the outer membrane and immediately converted to nucleosides by a 5′-nucleotidase. These nucleosides are then transported across the inner membrane by NupC. Once inside the cytosol, nucleosides are converted to bases and nucleotides. *De novo* purine biosynthesis uses ribose-5-P to generate the purine intermediate IMP in multiple steps requiring PurR-regulated enzymes. IMP can be converted to the purine nucleotides AMP or GMP. PRPP, phosphoribosyl pyrophosphate; APRT, adenine phosphoribosyltransferase; HPRT, hypoxanthine-guanine phosphoribosyltransferase; AICAR, 5-Aminoimidazole-4-carboxamide ribonucleotide.

Our understanding of *H. influenzae*'s purine metabolism and regulation derives from parallels with *Escherichia coli* and is shown schematically in Fig. 1. Extracellular nucleotides are converted to their respective nucleosides in the periplasm, and these are transported into the cytosol by NupC. In the cytosol, DeoD converts purine nucleosides to their respective bases; these can in turn be converted back into nucleotides by the HPRT and APRT phosphoribosyl transferases (Gots and Benson, 1973). Engodenous purine biosynthesis involves multiple steps to convert PRPP to the intermediate ribonucleoside monophosphate IMP, and is repressed by the master regulator PurR when extracellular nucleotides are available (Fig. 1). Aside from purine biosynthesis genes, PurR regulates genes for purine transport, salvage and interconversion pathways, and also downregulates the genes in pyrimidine biosynthetic and transport pathways (Ravcheev *et al*., 2002; Zhang *et al*., 2008; Cho *et al*., 2011). In the presence of guanine or hypoxanthine, *E. coli* PurR binds to a 16 bp palindromic sequence located within the promoter region of *pur* regulon genes (Cho *et al*., 2011). In *H. influenzae*, similar binding sites are found upstream of *pur* gene homologues (Mironov *et al*., 1999; Ravcheev *et al*., 2002). PurR's role in natural competence is not known, though Macfadyen *et al*. hypothesized that it might repress one or more competence genes to prevent DNA uptake when purines are abundant (Macfadyen *et al*., 2001). In this work we investigate the role of nucleotide availability on competence regulation and find that purine availability instead controls competence by controlling Sxy translation. We also show that nucleotide availability regulates competence in *Actinobacillus pneumoniae* and *A. suis*, relatives of *H. influenzae*, confirming the importance of such regulation.

## Results

### Extracellular purine nucleotides reduce Sxy translation

**Fig. 2:**
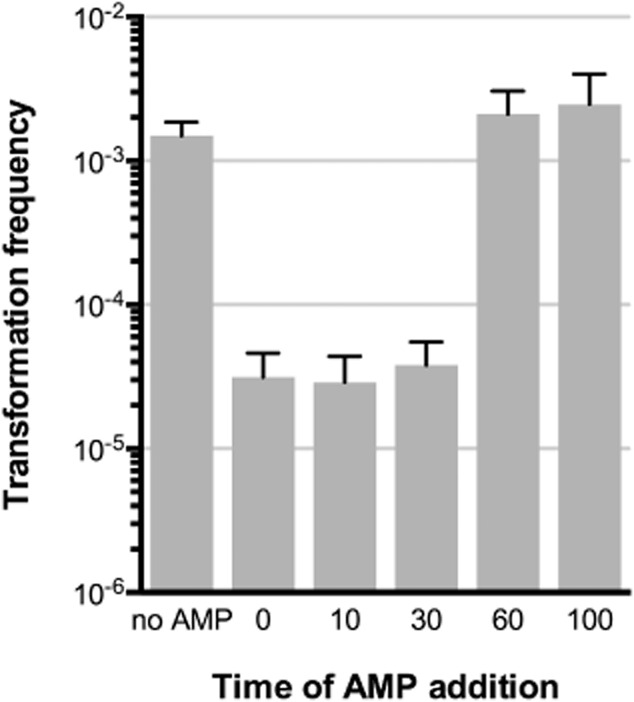
Effect of the timing of AMP addition to the inhibition of competence. sBHI-grown cells were transferred to MIV with 1 mM AMP added at different times during the MIV 100 min incubation. Transformation frequency was measured after 100 min. Each bar represents the mean of three biological replicates ± standard deviations.

**Fig. 3:**
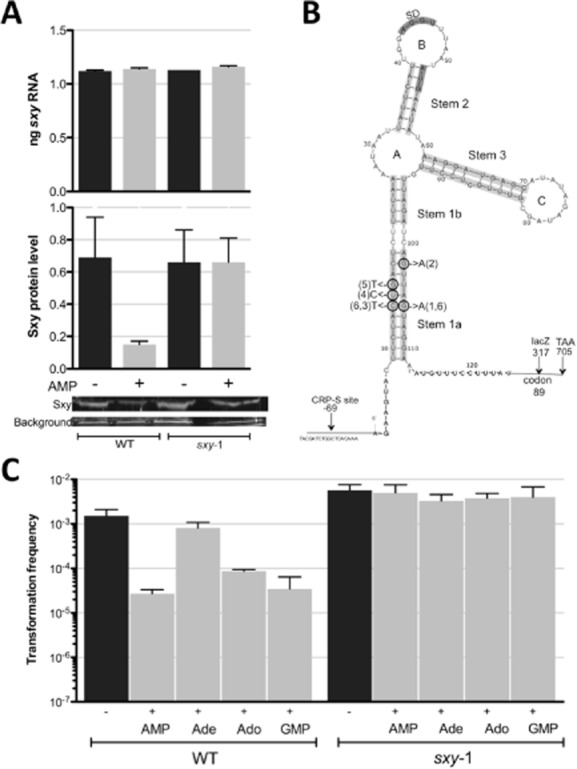
Purines repress Sxy expression unless the mRNA stem is weakened. A. qPCR (top) and western blot (bottom) analysis of *sxy* RNA and protein expression in the presence (grey bars) and absence (black bars) of 1 mM AMP after 100 min in MIV (see *Experimental procedures* for details). Error bars represent the mean of three biological replicates. Sxy protein levels are relative to the unidentified protein used for internal standardization in Cameron *et al*. (2008). A representative Western blot below the bottom graph shows Sxy protein and the standardization protein. WT, Wild type. B. Secondary structure of *sxy* mRNA. The stem structure, the position of individual mutations and other relevant sequence features are shown. C. sBHI-grown cells were transferred at OD_600_ 0.2 to MIV with (grey bars) or without (black bars) 1 mM purine source for 100 min before measuring transformation frequency (Ade = adenine, Ado = adenosine). Each bar represents the mean of three biological replicates ± standard deviations. WT, Wild type.

Macfadyen *et al*. showed that the addition of purine nucleotides reduced competence 100-fold (Macfadyen *et al*., 2001). They also showed that AMP addition reduced expression of two competence genes, suggesting that it acts on competence induction rather than later stages of competence. We confirmed this by showing that AMP addition only repressed competence when added as or shortly after cells were transferred to MIV, and that it had no effect when added at later time points when cells are already competent (Fig. 2). Macfadyen *et al*. also showed that AMP addition does not change the PTS/cAMP response to MIV (Macfadyen *et al*., 2001), so we hypothesized that it might affect the competence-specific activator Sxy. Western blotting showed that Sxy protein levels were severely reduced by the addition of AMP (Fig. 3A, bottom graph), suggesting that competence is low in the presence of purine nucleotides because Sxy levels are low. In contrast, qPCR showed that *sxy* mRNA was unchanged by the addition of AMP (Fig. 3A, top graph), suggesting that purine nucleotides specifically regulate Sxy translation.

**Fig. 4:**
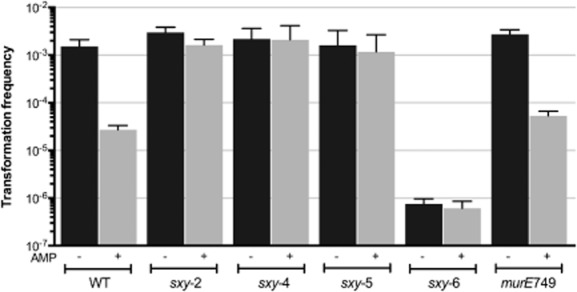
Competence is insensitive to purine addition in stem loop mutants. sBHI-grown cells were transferred at OD_600_ 0.2 to MIV with (grey bars) or without (black bars) 1 mM AMP for 100 min before measuring transformation frequency. Each bar represents the mean of three biological replicates ± standard deviations. WT, Wild type.

To determine whether the based-paired stem of *sxy* mRNA is the sensor that responds to purine nucleotides, we measured RNA and protein levels in the hypercompetent mutant *sxy*-1. In this mutant, a G-A transition at position 106 destabilizes the mRNA stem (Fig. 3B) and increases *sxy* translatability; analysis of compensatory mutations confirmed the essential role of base pairing (Redfield, 1991; Cameron *et al*., 2008). In contrast to wild type, *sxy*-1 cells maintained high levels of Sxy protein in MIV + AMP (Fig. 3A, bottom graph), and competence was unaffected by the addition of AMP or other purine sources (adenine, adenosine or GMP) (Fig. 3C). Together these results provide further evidence that purine nucleotides directly affect Sxy translation. Competence was also independent of AMP in the other available stem *sxy*-2-5 mutants (Fig. 4), whose mutations fall elsewhere in the *sxy* mRNA stem, but have the same destabilizing effect (Fig. 3B) (Cameron *et al*., 2008). The *sxy*-6 mutant, whose compensatory mutations restore base pairing in the stem and thus increase its stability (Cameron *et al*., 2008), served as a negative control (Figs 3B and 4). The *murE749* hypercompetent strain (Ma and Redfield, 2000) provided another important control because the mutation that makes it hypercompetent is in *murE* itself and not in the *sxy* stem. The sensitivity of *murE749* to AMP (Fig. 4) confirmed the specificity of the stem mutants' resistance to AMP. The insensitivity of mutants with destabilized stems to AMP is thus not simply attributable to higher levels of Sxy protein, and suggests that the *sxy* mRNA stem plays an important role in sensing and/or responding to nucleotide availability.

### PurR's role in purine nucleotide repression of competence is indirect

Macfadyen *et al*. found candidate PurR binding sites in the promoters of several CRP-S genes, suggesting that they may be repressed under conditions when PurR is active (Macfadyen *et al*., 2001). We therefore checked for this additional level of competence regulation by purine nucleotides.

The first step was to search for candidate PurR binding sites in the CRP-S promoters of competence gene. Using the *E. coli* PurR motif as a starting point, Ravcheev *et al*. developed species-specific scoring matrices for *H. influenzae* and several other gamma-proteobacteria (Ravcheev *et al*., 2002). We used their *H. influenzae* matrix to score the *H. influenzae* genome. Promoters with high-scoring sites include expected purine biosynthesis genes, as well as cytoplasmic and periplasmic phosphatases (Table 1). The only competence gene was *rec2* (score 5.31), whose product is required for translocation of ssDNA across the inner membrane (Barouki and Smith, 1985). The regions upstream of other *H. influenzae* CRP-S genes did not contain candidate PurR sites (highest score: 4.19, upstream of the *sxy* gene). Though the candidate *rec2* site had not been detected in previous studies (Mironov *et al*., 1999; Ravcheev *et al*., 2002), it is conserved in 20 sequenced *H. influenzae* genomes. However, such sites were not found upstream of *rec2* homologues in other Pasteurellaceae or gamma-proteobacteria (*E. coli*, *V. cholerae*), and recent ChIP and gene expression studies of *E. coli* did not identify *rec2* as a PurR target (Cho *et al*., 2011).

**Table 1:** Putative PurR binding sites in *H. influenzae.*

Score	Sequence	ORF	Position	Transcript	Process
6.00	ACGCAAACGTTTGCTT	HI0887	−63	*purH-purD-glyA*	purine biosynthesis
5.97	AAGCAAACGTTTGCTA	HI1726	−77	*hemH (purC)*	purine biosynthesis
5.85	AAGCAAACGTTTGCGA	HI1429	−63	*purM-purN*	purine biosynthesis
5.75	AAGCAAACGTTTGCAT	HI0752	1	*purL*	purine biosynthesis
5.74	AAGAAAACGTTTGCGT	HI1206	−82	*cvpA-purF*	purine biosynthesis
5.70	AGGCAAACGTTTGCTA	HI1615	−75	*purE-purK*	purine biosynthesis
5.68	AAGTAAACGTTTGCGT	HI0125	−83	*HI0125*	unknown
**5.31**	**ATGCAAACGGTTGCTT**	**HI0061**	−**70**	***rec2***	**natural competence**
5.27	TAGCAAACGCTTTCTT	HI0609	−45	*folD*	1-carbon metabolism
5.16	TTGCAAACGGTTGCTT	HI1736	−95	*HI1736*	unknown
5.11	TGGCAAACGATTGCTA	HI0495	−85	*aphA*	phosphatase, periplasmic
4.88	AAGTAAACGATTGTGT	HI0464	−34	*rpiA-serA*	purine biosynthesis
4.84	AGGAAAACGTTTCCGT	HI0638	−26	*hflD-purB*	purine biosynthesis
4.59	ATGCAAACGATTACTC	HI0667	−76	*glpX*	phosphatase,cytoplasmic
4.54	ATAAAATCGTTTGCTA	HI1153	−41	*hpt*	purine biosynthesis

The PurR-binding motif of Ravcheev *et al*. (2002) was used to score the *H. influenzae* Rd KW20 genome (see *Experimental procedures*). The sequences for ORFs on the reverse strand have been reverse complemented. The *rec2* site is shaded and bold. **ORF** indicates the gene nearest to the site. **Position** indicates the distance of the start codon from the distal end of the site (total site length = 16 bp). **Transcript** indicates the predicted genes regulated by the PurR site. **Process** indicates annotated metabolic processes encoded by the transcript. All 20 sequenced *H. influenzae* genomes had putative sites upstream of *rec2*, 18 with the same score as Rd KW20, and two strains (22.4–21 and PittEE) with better sites (site: ATGCAAACGTTTGCTT, score: 5.75).

**Fig. 5:**
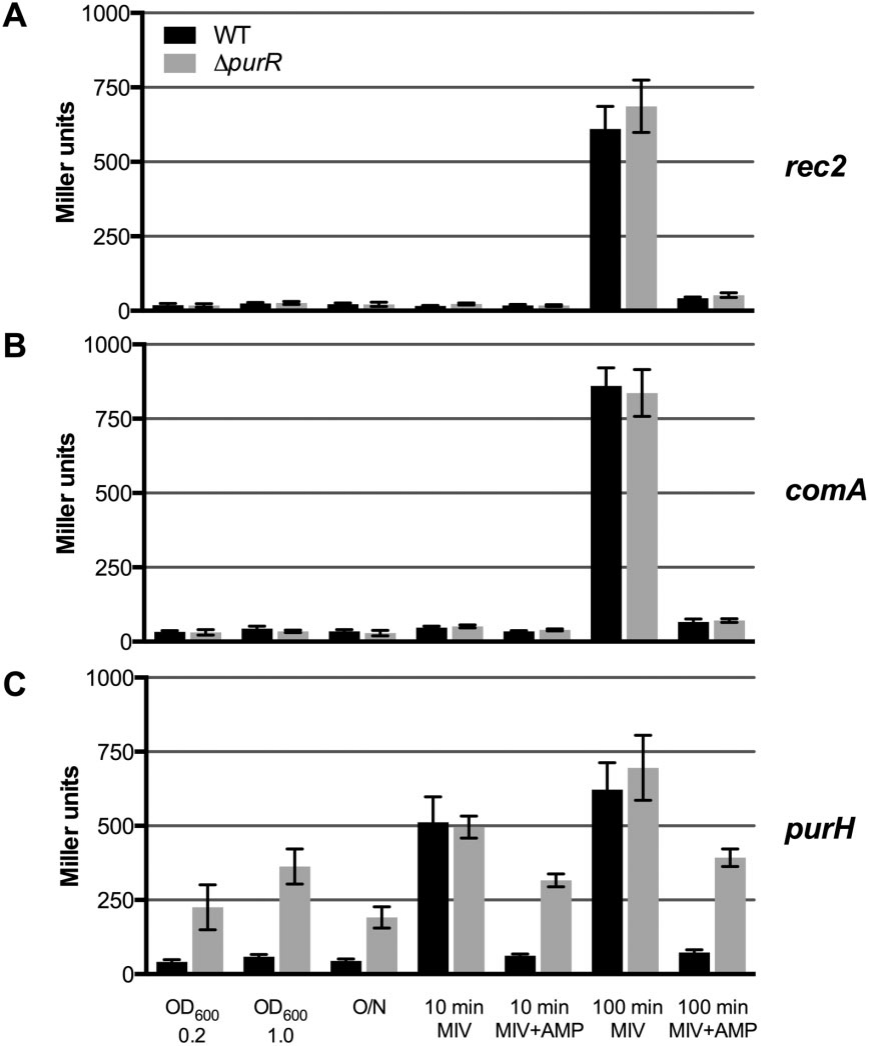
*rec2* expression is unchanged by the absence of PurR. Expression of the *H. influenzae* chromosomal *rec2::lacZ* (A), *comA::lacZ* (B) and *purH::lacZ* (C) fusions in the presence (black, WT: wild type) or absence (grey) of PurR. β-Galactosidase activity of the fusions under different conditions is shown as Miller units. Each bars represents the mean of three biological replicates ± standard deviations.

To directly test whether PurR represses *H. influenzae rec2*, we measured expression of a *rec2::lacZ* fusion in the presence and absence of PurR. We constructed an insertion deletion mutant of *purR*, replacing amino acids 5 to 299 of PurR with a kanamycin-resistance cassette, ensuring that both the DNA-binding and catalytic activities were disrupted. In this strain, purine biosynthesis genes are expected to be constitutively expressed at all growth stages. As negative and positive controls, we also tested expression of *lacZ* fusions to the CRP-S gene *comA* and to the PurR-regulated gene *purH*. As shown in Fig. 5A and B, deletion of PurR did not change *rec2* or *comA* expression, when CRP/Sxy expression was low (after overnight culture or in early log phase), intermediate (late log phase), or high (upon transfer to MIV). As expected, expression of *rec2* and *comA* remained low in cells transferred to AMP-supplemented MIV, and did not change upon deletion of *purR*. In contrast, *purH* was strongly induced by the absence of PurR under all conditions tested (Fig. 5C). A careful time-course monitoring *rec2* expression throughout growth in sBHI also showed no effect of *purR* deletion (data not shown). These results confirm that PurR does not repress *rec2*.

**Fig. 6:**
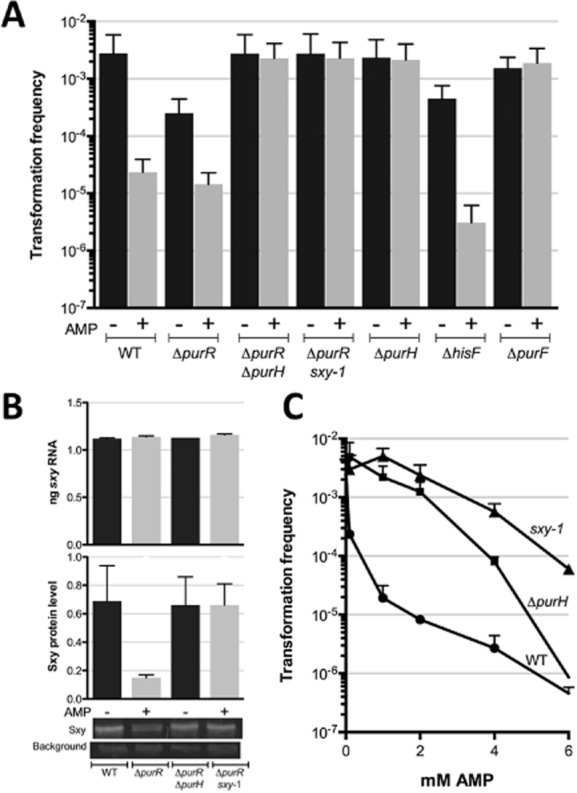
Natural competence in the presence and absence of AMP. sBHI-grown cells were transferred to MIV with (grey bars) or without (black bars) 1 mM AMP (A), or with increasing concentrations of AMP (C) for 100 min before measuring transformation frequency. Each bar or point represents the mean of three biological replicates ± standard deviations. Circles: WT (wild type), squares: Δ*purH*, triangles: *sxy*-1. B. qPCR (top) and western blot (bottom) analysis of *sxy* RNA and protein expression in the presence (grey bars) and absence (black bars) of 1 mM AMP after 100 min in MIV (see *Experimental* procedures for details). Error bars represent the mean of three biological replicates. Sxy protein levels are relative to the unidentified protein used for internal standardization in Cameron *et al*. (2008). A representative Western blot below the bottom graph shows Sxy protein and the standardization protein.

One further test was to assess competence of the *purR*^−^ strain in the presence of purines: if purine nucleotides' repressing effect on competence is mediated through PurR, the effect should disappear in the *purR*^−^ strain. As shown in Fig. 6A, the addition of AMP still reduced competence in the *purR*^−^ strain. These results in combination with those of Fig. 5 confirm that purine repression of competence is not mediated by PurR's repression of competence genes.

### Increased intracellular purine nucleotides repress *sxy* expression by blocking translation

Figure 6A shows that not only was competence not raised in cells lacking *purR*, it was lowered ∼ 10-fold; this was not due to differences in growth of the mutant strain (Fig. S2). Cells lacking PurR are expected to have elevated intracellular purine nucleotide pools due to constitutive expression of purine biosynthesis genes, so we hypothesized that the reduced competence of the *purR*^−^ strain might have the same cause as that of wild-type cells treated with extracellular purine nucleotides.

To test whether elevated purine pools cause the competence defect of the *purR^−^* strain, we eliminated endogeneous purine biosynthesis in this strain by further knocking out *purH*, whose product converts AICAR into IMP, the last common precursor of AMP and GMP (Fig. 1). In this strain, all purine biosynthesis genes are constitutively expressed but purines cannot be synthesized. As shown in Fig. 6A, the *purH*^−^ mutation restored competence of the *purR*^−^ strain to wild-type levels, confirming our hypothesis. Next, we measured Sxy protein levels and found them to be significantly reduced in the *purR*^−^ strain, and similar to wild type in the *purR*^−^ *purH*^−^ strain (Fig. 6B). As expected, *sxy* mRNA levels were similar in all strains (Fig. 6B). Finally, because mutations that weaken the *sxy* mRNA stem overcome the inhibitory effect of high extracellular purines, we tested whether they could also overcome the competence defect of the *purR*^−^ strain. As shown in Fig. 6A, the competence defect of the *purR*^−^ strain was abolished by the *sxy-*1 mutation. Collectively these results confirm that increased purine nucleotide pools, whether extracellular or intracellular, reduce competence by blocking Sxy translation.

Because the removal of endogeneous purine biosynthesis overcame the repression of competence by high intracellular purine pools (*purR^−^ purH^−^* strain), we tested its effect on the repression of competence by extracellular AMP. As shown in Fig. 6A, addition of the *purH*^−^ mutation made the *purR*^−^ strain insensitive to 1 mM AMP. Surprisingly, a *purR*^+^ *purH*^−^ strain was also insensitive to AMP (Fig. 6A), suggesting that the repression of competence by AMP is overcome by the accumulation of intermediates in the purine biosynthesis pathway upstream of PurH. In *Salmonella enterica*, *purH*^−^ cells have increased PPRP pools due to feedback inhibition of *purF* and other upstream purine biosynthesis genes by AICAR (Allen *et al*., 2002; Bazurto and Downs, 2011). Since PRPP is needed to convert bases to nucleotides, it is possible that cells lacking *purH* are better able to convert extracellular purines. To test whether the phenotype of the *purH*^−^ strain is attributed to increased PRPP pools, we created a knockout in *purF*. PurF carries out the first step in the purine biosynthetic pathway, the conversion of PPRP (phosphoribosyl pyrophosphate) to ribosylamine-5P (Fig. 1). We also created a knockout of *hisF*, a cyclase enzyme required for the last step of histidine metabolism that also produces AICAR (Fig. 1). We found that competence was still inhibited by AMP in cells lacking *hisF*, while cells lacking *purF*, like those lacking *purH*, were insensitive to extracellular AMP addition (Fig. 6A). This confirms that increased intracellular PRPP pools enable competent cells to overcome the inhibitory effects of extracellular purine nucleotides. Because the inhibition of competence by purine nucleotides is concentration-dependent (Macfadyen *et al*., 2001), we tested whether AMP at higher concentrations would overcome the higher PRPP pools of *purH^−^* cells and inhibit competence. As shown in Fig. 6C, competence was inhibited by AMP concentrations as low as 0.1 mM in wild-type cells, whereas comparable inhibition was only seen upwards of 4 mM AMP in *purH*^−^ cells. In contrast, inhibition of the *sxy*-1 strain occurred only at concentrations high enough to significantly affect viability (Figs 6C and S1).

### Purine regulation of Sxy translation is indirect

**Fig. 7:**
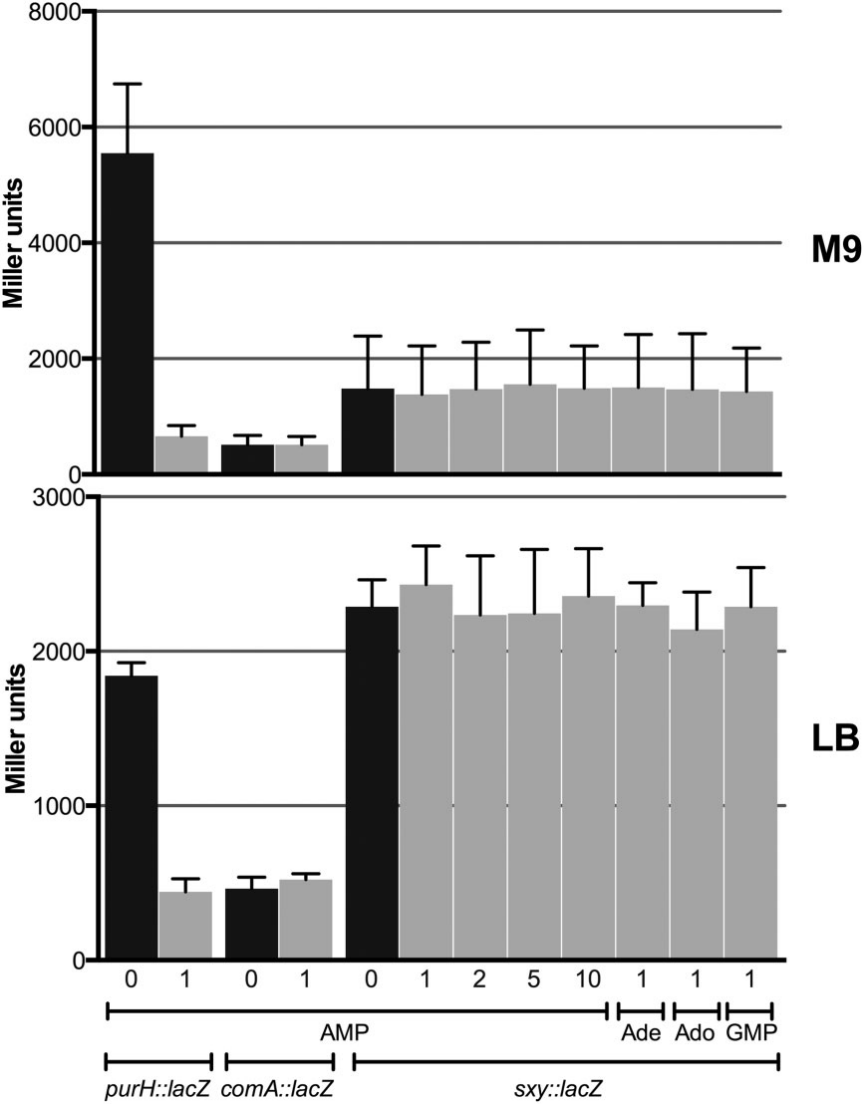
The Sxy stem loop does not directly respond to purines. The expression of plasmid-encoded fusions in *E. coli* was measured in the presence (grey bars) or absence (black bars) of purine sources, after 30 min in M9 minimal medium (top graph) or LB (bottom graph). The β-galactosidase activity from the translational *sxy::lacZ* fusion, and transcriptional *comA::lacZ* and *purH::lacZ* fusions is shown as Miller units. The concentrations of AMP are given below each bar in mM; adenine (Ade) and adenosine (Ado) were used at 1 mM. Each bar represents the mean of three biological replicates ± standard deviations.

The above results suggest that an increase in intracellular purine pools limits Sxy translation and competence. The simplest model is that cytosolic purine intermediates directly bind to the *sxy* mRNA stem, and stabilize it to repress translation. Testing whether the Sxy stem itself responds to purines is complicated by additional levels of regulation present in *H. influenzae*. Thus we instead measured the expression of a translational *sxy::lacZ* fusion in the presence and absence of purine sources in *E. coli*. In this fusion, the *lacZ*::kan cassette is fused to codon 89 of the *H. influenzae sxy* coding sequence, leaving the stem intact (Fig. 3B) (Cameron *et al*., 2008). As positive and negative controls, we used plasmid-encoded versions of the transcriptional *lacZ* fusions to *H. influenzae purH* and *comA* described above. We tested expression of all fusions both in rich and minimal media. As expected, the expression of *purH* was lowered by AMP addition in both media (Fig. 7). The difference in expression between these media likely reflects inhibition of *purH* expression by abundant purines in the Luria–Bertani (LB) medium. Also as expected, the expression of the negative control *comA* was the same in both media and was unchanged by AMP addition (Fig. 7). The expression from the *sxy* stem was high [two- to fivefold higher than that seen under competence-inducing conditions in *H. influenzae* (Cameron *et al*., 2008)], but was not changed by AMP even at higher concentrations, or by GMP, adenine or adenosine (Fig. 7). This suggests that exogeneous purine nucleotides do not limit Sxy translation in *E. coli*.

### Purine regulation of Sxy translation does not require Hfq

We next tested whether Sxy translation was regulated by a small regulatory or antisense RNA, which would respond (directly or indirectly) to purine levels. Translation of the *V. cholerae sxy* homologue is activated by the global RNA regulator Hfq and the small RNA *tfoR* (Yamamoto *et al*., 2011). Moreover, Cameron *et al*. showed that such regulation is possible in *H. influenzae*, as a ssDNA oligo designed to be complementary to part of *H. influenzae sxy* mRNA competed with stem formation *in vitro* and increased translation (Cameron *et al*., 2008).

**Fig. 8:**
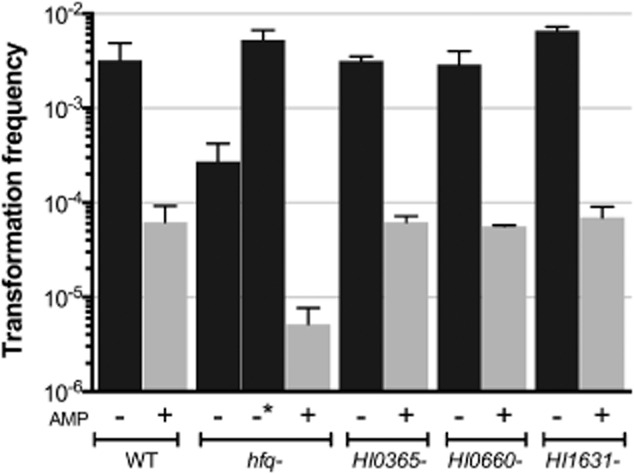
AMP inhibits competence in mutants of *hfq*, *HI0365*, *HI0660* and *HI1631*. sBHI-grown cells were transferred at OD_600_ 0.2 to MIV with (grey bars) or without (black bars) 1 mM AMP for 100 min before measuring transformation frequency. Each bars represents the mean of at least two biological replicates ± standard deviations. WT, Wild type; *hfq^−^ *sxy*-1.

To test whether Hfq is involved in regulation of *sxy* expression in *H. influenzae*, we examined the effect on competence of a deletion of *hfq*. Competence of the *hfq*^−^ strain was reduced about 10-fold, a defect that was overcome by the *sxy-*1 stem mutation (Fig. 8), suggesting that Hfq may participate in the unfolding of the stem to enable translation. However, competence was still strongly repressed by AMP in the *hfq*^−^ strain by the same factor as wild-type cells (Fig. 8), indicating that if Hfq participates in *sxy* regulation, it does not do so by sensing purine pools.

### Purine regulation of Sxy translation does not require HI0365, HI0660 or HI1631

We recently characterized the competence phenotypes of every CRP-S gene (Sinha *et al*., 2012). Several have no role in the DNA uptake process and lack any characterized functions, so we tested whether any of these might affect competence regulation in the presence of purine nucleotides through feedback regulation of Sxy translation. As shown in Fig. 8 (right bars), the competence of knockouts of HI0365, HI0660 and HI1631 was still repressed by AMP, confirming that their products do not affect purine regulation of competence.

### The impact of purine nucleotides on competence is not limited to *H. influenzae*

We examined whether high purine pools also prevent competence in relatives of *H. influenzae*, by testing the effect of AMP addition in two other naturally competent Pasteurellacean species, *Actinobacillus pleuropneumoniae* and *Actinobacillus suis* (Redfield *et al*., 2006; Maughan *et al*., 2008). Natural competence is known to be induced by starvation and dependent on Sxy/CRP in *A. pleuropneumoniae* (Bosse *et al*., 2009), but has not been studied in *A. suis*.

**Fig. 9:**
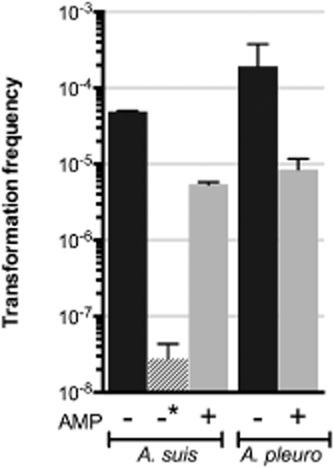
AMP inhibits competence in *Actinobacillus pleuropneumoniae* (*A. pleuro*) HS143 and *A. suis* ATCC15557. sBHI-grown cells were transferred at OD_600_ 0.2 to MIV with (grey bars) or without (black bars) 0.1 mM AMP for 100 min before measuring transformation frequency. Each bar represents the mean of three biological replicates ± standard deviations. *A. suis Δ*sxy*. The hashed bar represents the limit of detection of the *sxy*− strain.

We first demonstrated that *A. suis* is naturally competent in the starvation medium used to induce competence in other Pasteurellaceae, and found that this is entirely dependent on Sxy (Fig. 9). In addition, like other Pasteurellaceae, the *A. suis* H91-0380 genome contains homologues of essential competence gene, with conserved CRP-S sites in their promoters (Table 2). As shown in Fig. 9, competence was reduced by addition of AMP in both species, suggesting that the mechanisms by which AMP regulates competence are conserved. Since *A. pleuropneumoniae* and *A. suis* are both distant relatives of *H. influenzae*, we conclude that purine nucleotide repression of Sxy translation is likely to be an ancestral property of the Pasteurellaceae.

**Table 2:** Competence genes homologues and their CRP-S site in the sequenced strain of *A. suis* H91-0380

Score	Sequence	Position	Transcript	Start	End
32.0	TTTTGCGATCCTGATCGAAAAA	−87	*comNOPQ*	1786332	1788485
31.3	TTTTGCGATCTGGATCGCAAAC	−83	*rec2*	675504	677798
30.9	TTTTGCGATCTTGATCGAAAAT	−165	*HI0365/pilF2*	82751	84535
30.6	TTTTTCGATCTATATCGCAAAA	−76	*comF*	2430643	2431320
30.4	TTTTGCGATCAAGATCGAGAAA	−99	*comE1*	(2267009)	(2267374)
30.2	TTTTGCGATCCTGATCGAGAAA	−87	*comM*	2145444	2146973
29.8	TTTTTCGATGAAGATCGCAAAA	−133	*comABCDE*	(1349840)	(1353261)
29.7	TTTTGTGATCTCAATCGAAAAA	−181	*dprA*	2097055	2098209
28.0	TTTTTCGACGCAGATCGCAAAA	−78	*radC*	(1640152)	(1640826)
27.1	ATTCTCGATCCGGATCGCAAAA	−85	*pilABCD*	(1114637)	(1118340)
18.4	TTTCAGGTAGAATGTCGCACAA	−57	*ssb*	986417	986953
16.4	TTGTTGCATCATTTCGCCAAAA	−96	*HI0569/60*	2413794	2413813

The sequences for ORFs on the reverse strand have been reverse complemented. **Position** indicates the distance of the start codon from the distal end of the site. **Transcript** indicates the predicted genes regulated by the CRP-S site. **Start** and **End** indicate the coordinates of the transcript.

## Discussion

Twenty years ago, we hypothesized that competent cells take up DNA primarily for its nucleotides, and predicted that competence genes would be induced by nucleotide limitation (Redfield, 1993). In this work, we show that extracellular purine nucleotides specifically reduce expression of the competence regulator Sxy, by inhibiting its translation. This reduction does not involve Hfq-dependent small RNA processes or three competence-regulated proteins of unknown function. Expression of a *sxy::*lacZ translational fusion was unaffected by extracellular purine intermediates in *E. coli.* Moreover, the *sxy* stem structure bears no resemblance to known purine riboswitches and preliminary analyses have shown that *sxy* mRNA does not bind purine compounds in a riboswitch assay. This suggests an alternative model of regulation, where one or more purine-binding proteins sense purine levels in the cytoplasm and activate or repress regulators of Sxy translation. Further work is required to determine if such regulators exist and how they act.

We have shown that nucleotide abundance also regulates competence in relatives of *H. influenzae* that inhabit similar niches in different hosts: *H. influenzae* colonizes the human nasopharynx and middle ear, while *A. pleuropneumoniae* and *A. suis* colonize the upper respiratory tracts of pigs. DNA is abundant in both niches, as it is in many mucosal environments, and can serve as a rich nutrient source. The importance of DNA as a nutrient and its involvement in competence regulation has also been demonstrated in other families of the gamma-proteobacteria. For example, Finkel and co-workers showed that *E. coli* could grow by using DNA as the sole source of carbon and energy, and that this required homologues of *H. influenzae*'s competence genes (Finkel and Kolter, 2001; Palchevskiy and Finkel, 2006). We have shown that these homologues are required for DNA uptake by *E. coli*, though conditions that naturally induce this process are not known (Sinha and Redfield, 2012). The transfer to starvation conditions does not induce *E. coli sxy* expression, suggesting that other signals are likely required (Sinha *et al*., 2009; Sinha and Redfield, 2012). Another example of nucleotide abundance regulating competence comes from *V. cholerae*, whose competence is inhibited by cytidine (Antonova *et al*., 2012). This was attributed to disrupted interactions between CRP and the nucleoside-scavenging cytidine repressor CytR. *H. influenzae* lacks a homologue of CytR, so the mechanisms of nucleotide inhibition are different in both organisms. Though Macfadyen *et al*. found no effect of pyrimidine nucleotides on *H. influenzae* competence (Macfadyen *et al*., 2001), it is possible that an inhibitory effect exists *in vivo*. *H. influenzae* cannot synthesize pyrimidines *de novo* and relies instead on salvage from the medium of pyrimidines or precursors. Pyrimidine pools thus never get depleted under our standard laboratory conditions, because they are abundant in rich medium (sBHI) and because the MIV medium used to induce competence contains the pyrimidine precursor citrulline.

All Pasteurellaceae *sxy* genes are preceded by long intergenic regions that could form into stem structures like those of *H. influenzae sxy* and *V. cholerae tfoX*, and contribute to regulation. At present, most transcriptional start sites have not yet been mapped, which prevents predictions of stem structures and more detailed studies of regulation. Like the *V. cholerae tfoX* stem, the *H. influenzae sxy* stem may integrate multiple signals to regulate natural competence: aside from purine availability, it might also respond to separate signals through Hfq. Further work is required to confirm whether Hfq regulates *sxy* expression, as well as to identify the conditions under which regulation occurs and the small RNA(s) involved. A search of the genome for small regulatory RNAs using RNAPredator (Eggenhofer *et al*., 2011) or the reverse search mode of TargetRNA (http://snowwhite.wellesley.edu/targetRNA/index_2.html) found some sequences with complementarity to *sxy* mRNA, but these were often short or showed modest identity. In contrast, performing the same search with the *V. cholerae tfoX* sequence identified *tfoR* within the top matches (and a search with *tfoR* identified *tfoX*). The best chance of identifying comparable small RNAs in *H. influenzae* would be to screen genomic libraries for clones that change *sxy* expression as was done to identify *tfoR* (Yamamoto *et al*., 2011).

This work dissects the regulation of a biological process to inform an understanding of its evolutionary function. DNA is abundant in the human nasopharynx where *H. influenzae* lives, and we have demonstrated the importance of intracellular nucleotide depletion for competence induction, through the competence regulator Sxy. Because *sxy* transcription requires CRP (Cameron *et al*., 2008), the nucleotide depletion and sugar depletion signals are tightly linked. Further competence-inducing signals may exist as in other bacteria, and each clarifies the conditions under which DNA uptake is favourable.

## Experimental procedures

### Bacterial strains, plasmids and culture conditions

The strains and plasmids used in this study are listed in Table 3. *Escherichia coli* was cultured at 30°C or 37°C in LB medium, with ampicillin (100 μg ml^−1^) and spectinomycin (40 μg ml^−1^) when required. *H. influenzae* was cultured at 37°C in brain heart infusion (BHI) medium supplemented with NAD (2 mg ml^−1^) and hemin (10 mg ml^−1^) (sBHI), with novobiocin (2.5 μg ml^−1^), spectinomycin (20 μg ml^−1^), chloramphenicol (2 μg ml^−1^) or kanamycin (7 μg ml^−1^) when required. *Actinobacillus pleuropneumoniae* was cultured at 37°C on BHI agar supplemented with 10% Levinthal's Base with or without 50 μg ml^−1^ kanamycin, or in BHI broth supplemented with 100 μg ml^−1^ β-nicotinamide adenine dinucleotide (NAD). *A. suis* was obtained from Janet MacInnes and cultured at 37°C in plain BHI medium, with or without 25 μg ml^−1^ nalidixic acid.

**Table 3:** Bacterial strains and plasmids

Name	Organism	Genotype	References/notes
Bacterial strains			
HS143	*A. pleuropneumoniae*	WT	Bosse *et al*. (2009)
4074 Δ*sodC*	*A. pleuropneumoniae*	*sodC::*kan	Bosse *et al*. (2009); donor DNA for transformation assays
ATCC 15557	*A. suis*	WT	Wetmore *et al*. (1963)
ATCC 15557 Δ*sxy*	*A. suis*	*sxy::*spc	This study
SO4 nal^R^	*A. suis*	nal^R^	Janet MacInnes; donor DNA for transformation assays
DH5α	*E. coli*	*F80lacZ Δ(lacIZYA-argF) endA1*	Taylor *et al*. (1993); used for routine cloning
SW102	*E. coli*	F- *mcrA* Δ(*mrr-hsdRMS*-*mcrBC*) Φ80d*lacZ*ΔM15 Δ*lacX*74 *deoR recA*1 end*A*1 *araD*139 Δ(*ara*, *leu*)7649 *rpsL nupG* [*λcI*857 (*cro-bio*A) <> *tet*] Δ*galK*	Warming *et al*. (2005); used for recombineering
KW20	*H. influenzae*	WT	Alexander and Leidy (1951)
MAP7	*H. influenzae*	str^R^ kan^R^ nov^R^ nal^R^ spc^R^ vio^R^ stv^R^	Barcak *et al*. (1991); donor DNA for transformation assays
Δ*purR*	*H. influenzae*	KW20 *purR::*kan	This study
Δ*purR2*	*H. influenzae*	KW20 *purR::*spc	This study
Δ*purH*	*H. influenzae*	KW20 *purH::*spc	This study
Δ*purR purH*	*H. influenzae*	KW20 *purR::*kan *purH::*spc	This study
*rec2::*lacZ	*H. influenzae*	KW20 *rec2::*lacZ-cm (operon fusion)	Gwinn *et al*. (1997)
*comA::*lacZ	*H. influenzae*	KW20 *comA::*lacZ-cm (operon fusion)	Gwinn *et al*. (1997)
*purH::*lacZ	*H. influenzae*	KW20 *purH::*lacZ-kan (operon fusion)	This study
Δ*purR rec2*:lacZ	*H. influenzae*	KW20 *rec2::*lacZ-cm (operon fusion) *purR::*kan	This study
Δ*purR comA*::lacZ	*H. influenzae*	KW20 *comA::*lacZ-cm (operon fusion) *purR::*kan	This study
Δ*purR purH*::lacZ	*H. influenzae*	KW20 *purH::*lacZ-kan (operon fusion) *purR::*spc	This study
*sxy*-1	*H. influenzae*	KW20 *sxy*G_106_A	Redfield (1991)
*sxy*-2	*H. influenzae*	KW20 *sxy*G_102_A	Cameron *et al*. (2008)
*sxy*-4	*H. influenzae*	KW20 *sxy*T_15_C	Cameron *et al*. (2008)
*sxy*-5	*H. influenzae*	KW20 *sxy*G_16_T	Cameron *et al*. (2008)
*sxy*-6	*H. influenzae*	KW20 *sxy*C_14_T,G_106_A	Cameron *et al*. (2008)
*murE749*	*H. influenzae*	KW20 *murE*G_13033_A	Ma and Redfield (2000)
Δ*hfq*	*H. influenzae*	KW20 *hfq::*spc	This study
Δ*hfq sxy*-1	*H. influenzae*	KW20 *hfq::*spc *sxy*G_106_A	This study
Δ*HI0365*	*H. influenzae*	KW20 *HI0365::*spc	Sinha *et al*. (2012)
Δ*HI0660*	*H. influenzae*	KW20 *HI0660::*spc	Sinha *et al*. (2012)
Δ*HI1631*	*H. influenzae*	KW20 *HI1631::*spc	Sinha *et al*. (2012)
rpoB*	*H. influenzae*	KW20 rpoB*	This study
Plasmids			
p*comA*::lacZ	/	*comA::*lacZ-kan in pGEMT-Easy (operon fusion)	This study
p*purH*::lacZ	/	*purH::*lacZ-kan in pGEMT-Easy (operon fusion)	This study
pLBSF2	/	*sxy*_89_::lacZ-kan in pGEM (protein fusion)	Cameron *et al*. (2008)

Unless otherwise stated, AMP, adenine, adenosine, GMP and AICAR were used at final concentrations of 1 mM for *H. influenzae* and *E. coli*, and 0.1 mM for *A. pleuropneumoniae* and *A. suis*. These concentrations do not affect cell viability (Fig. S1).

### Strain construction and cloning

The sequences of primers used are given in Table S1. All strains were verified by PCR and sequencing. Mutations were transferred to the chromosome of *A. suis* or *H. influenzae* by natural transformation of MIV-competent cells (see below).

KW20 Δ*purR/*KW20 Δ*purR sxy*-1: A 3 kb region flanking and including *purR* was PCR-amplified and cloned into pGEMT-Easy (Promega) to generate pG*purR*. Inverse PCR on pG*purR* amplified a fragment lacking sequences between the first 32 bp and last 132 bp of *purR*, with *Kpn*I and *Eco*RI restriction enzymes sites added to the forward and reverse primers respectively. The PCR fragment was digested, and ligated to a *Kpn*I/*Eco*RI-digested kan^R^ cassette from pUC4K (Taylor and Rose, 1988). Positive clones (pG*purR::*kan) transformed into DH5α were isolated on medium containing ampicillin and kanamycin. The insert was PCR amplified using the universal primers M13F and M13R (Promega) and used for transformation into *H. influenzae* KW20 and *sxy*-1 cells. A second Δ*purR* strain (Δ*purR*2, *purR::*spc) was constructed in KW20 in the same way, but with spc^R^ from pRSM2832 (Tracy *et al*., 2008) replacing kan^R^, and using blunt-end cloning instead of *Kpn*I/*Eco*RI.

KW20 Δ*purH:* The strategy used to construct Δ*purR* was followed, with the following differences: the region flanking and including *purH* in pG*purH* was 4 kb, and the amplified inverse PCR fragment lacked sequences between the first 229 bp and last 442 bp of *purH* and contained *Hind*III and *Eco*RI sites. This was ligated to a *Hind*III/*Eco*RI-digested spc^R^ cassette from pRSM2832 (Tracy *et al*., 2008) to generate pG*purH::*spc.

KW20 Δ*purR purH:* The insert from pG*purH::*spc was used to transform competent cells of Δ*purR::*kan. Double transformants were selected on medium containing kanamycin and spectinomycin.

KW20 Δ*hfq/*KW20 Δ*hfq sxy*-1: The *hfq::*spec strain was constructed by recombineering using the procedure described in Sinha *et al*. (2012), replacing the sequence between the first and last seven codons of the *hfq* coding sequence with a spc^R^ cassette pRSM2832 (Tracy *et al*., 2008). This mutation was transferred to KW20 and to *sxy-*1 cells.

p*purH::lacZ* and p*comA::lacZ*: A 4 kb region flanking and including *purH* was PCR-amplified and ligated into pGEM-Easy (Promega) to generate plasmid pG*purH*. This plasmid was linearized with *Cla*I, blunt-ended using Klenow and ligated to a blunt *lacZ*-kan cassette excised from pLKZ83 with *Bam*HI (Barcak *et al*., 1991). The same strategy was followed with pG*comA-E* (Sinha *et al*., 2012) which was linearized with *Bgl*II and directly ligated to the BamHI-digested *lacZ* cassette. Clones in the forward orientation were selected on medium containing ampicillin, kanamycin and Xgal. The *purH::lacZ* insert and the *comA::lacZ* and *rec2::lacZ* fusions of Gwinn *et al*. (1997) were also transferred to KW20 and to Δ*purR* by natural transformation.

ATCC 15557 *Δsxy:* This *sxy::*spec derivative was constructed by recombineering using the procedure described in Sinha *et al*. (2012), replacing the sequence between the first and last seven codons of the *sxy* coding sequence in strain ATCC 15557 with a spc^R^ cassette pRSM2832 (Tracy *et al*., 2008).

### Transformation assays

*Haemophilus influenzae* cells were made competent by transfer of sBHI-grown cells to MIV medium at OD_600_ 0.2–0.25, and incubation for 100 min at 37°C as described previously (Poje and Redfield, 2003). When testing the effect of purine repression, AMP and other purine sources were added to MIV with the cells.

To measure transformation, competent cells were incubated with 1 μg of MAP7 chromosomal DNA in 1 ml of MIV culture for 15 min at 37°C, after which the free DNA was degraded by incubation with DNase I (10 μg ml^−1^) for 5 min. Cells were then diluted and plated on sBHI agar with and without novobiocin. Transformation frequencies were calculated by dividing the number of novobiocin-resistant colony-forming units (cfu) by the total number of cfu. Transformation in rich medium was measured in the same way, after adding 1 μg DNA to 1 ml culture aliquots.

### Search for PurR binding sites

Candidate PurR binding sites were identified in the *H. influenzae* Rd KW20 genome using the species-specific position weight matrix of Ravcheev *et al*. (2002). Scoring used custom R scripts, including the SeqInR add-on package to manipulate sequences (Charif and Lobry, 2007). Searches using *E. coli* PurR motifs (Mironov *et al*., 1999; Cho *et al*., 2011) on the *H. influenzae* genome found the same sites. A cutoff score of 4.5 captured all of the PurR-regulated genes identified by Ravcheev *et al*. (2002). An additional requirement of location between −100 and +20 of annotated start codons eliminated false-positive sites outside of promoter regions. blast was used to identify *rec2* homologues in other sequenced *Haemophilus influenzae*, Pasteurellaceae and selected gamma-proteobacteria, and the highest scoring PurR site from −200 to +20 of the start was determined.

### Search for CRP-S homologues in *A. suis*

Competence genes in the *A. suis* H91-0380 genome sequence were first identified by blast using *A. pleuropneumoniae* L20 homologues (Bosse *et al*., 2009). Next, their upstream regions were searched for candidate CRP-S binding sites, using a scoring matrix produced using the method of Schneider *et al*. (1986) from the set of Pasteurellaceaen CRP-S sites identified by Cameron and Redfield (2006).

### β-Galactosidase assays

*Haemophilus influenzae* strains containing chromosomal *lacZ* fusions were grown in sBHI or transferred to MIV as described above. *E. coli* strains containing plasmid-encoded *lacZ* fusions were grown in LB or in M9 with 0.4% glucose. Cultures were incubated with or without an added purine source and sampled at regular time intervals. After sampling, cells were immediately assayed for OD_600_ and pelleted by centrifugation, supernatants were removed and cell pellets were frozen at −20°C for later assays of β-galactosidase activity following the protocol of Miller (1992).

### Quantitative measurements of *sxy* expression

Real-time qPCR was used to measure *sxy* transcript levels, while Western blotting was used to measure Sxy protein levels. All procedures were carried out as described in Cameron *et al*. (2008), in which the primers and antibody used are also described. For each sample, *sxy* RNA levels were adjusted using *murG* levels, while Sxy protein levels were adjusted using an unidentified protein of constant abundance (Cameron *et al*., 2008) (‘background’ in Fig. 3).

## References

[b2] Alexander HE, Leidy G (1951). Determination of inherited traits of *H. influenzae* by desoxyribonucleic acid fractions isolated from type-specific cells. J Exp Med.

[b3] Allen S, Zilles JL, Downs DM (2002). Metabolic flux in both the purine mononucleotide and histidine biosynthetic pathways can influence synthesis of the hydroxymethyl pyrimidine moiety of thiamine in *Salmonella enterica*. J Bacteriol.

[b5] Antonova ES, Hammer BK (2011). Quorum-sensing autoinducer molecules produced by members of a multispecies biofilm promote horizontal gene transfer to *Vibrio cholerae*. FEMS Microbiol Lett.

[b4] Antonova ES, Bernardy EE, Hammer BK (2012). Natural competence in *Vibrio cholerae* is controlled by a nucleoside scavenging response that requires CytR-dependent anti-activation. Mol Microbiol.

[b6] Barcak GJ, Chandler MS, Redfield RJ, Tomb JF (1991). Genetic systems in *Haemophilus influenzae*. Methods Enzymol.

[b7] Barouki R, Smith HO (1985). Reexamination of phenotypic defects in *rec-1* and *rec-2* mutants of *Haemophilus influenzae* Rd. J Bacteriol.

[b8] Bazurto JV, Downs DM (2011). Plasticity in the purine-thiamine metabolic network of Salmonella. Genetics.

[b9] Blokesch M (2012). Chitin colonization, chitin degradation and chitin-induced natural competence of *Vibrio cholerae* are subject to catabolite repression. Environ Microbiol.

[b10] Bosse JT, Sinha S, Schippers T, Kroll JS, Redfield RJ, Langford PR (2009). Natural competence in strains *of Actinobacillus pleuropneumoniae*. FEMS Microbiol Lett.

[b11] Cameron AD, Redfield RJ (2006). Non-canonical CRP sites control competence regulons in *Escherichia coli* and many other gamma-proteobacteria. Nucleic Acids Res.

[b12] Cameron AD, Redfield RJ (2008). CRP binding and transcription activation at CRP-S sites. J Mol Biol.

[b13] Cameron AD, Volar M, Bannister LA, Redfield RJ (2008). RNA secondary structure regulates the translation of *sxy* and competence development in *Haemophilus influenzae*. Nucleic Acids Res.

[b14] Charif D, Lobry JR (2007). Seqin{R} 1.0-2: A Contributed Package to the {R} Project for Statistical Computing Devoted to Biological Sequences Etrieval and Analysis. in Structural Approaches to Sequence Evolution: Molecules, Networks, Populations.

[b15] Cho BK, Federowicz SA, Embree M, Park YS, Kim D, Palsson BO (2011). The PurR regulon in *Escherichia coli* K-12 MG1655. Nucleic Acids Res.

[b16] Domingues S, Harms K, Fricke WF, Johnsen PJ, da Silva GJ, Nielsen KM (2012). Natural transformation facilitates transfer of transposons, integrons and gene cassettes between bacterial species. PLoS Pathog.

[b17] Dorocicz IR, Williams PM, Redfield RJ (1993). The *Haemophilus influenzae* adenylate cyclase gene: cloning, sequence, and essential role in competence. J Bacteriol.

[b18] Eggenhofer F, Tafer H, Stadler PF, Hofacker IL (2011). RNApredator: fast accessibility-based prediction of sRNA targets. Nucleic Acids Res.

[b19] Finkel SE, Kolter R (2001). DNA as a nutrient: novel role for bacterial competence gene homologs. J Bacteriol.

[b20] Gots JS, Benson CE (1973). Genetic control of bacterial purine phosphoribosyltransferases and an approach to gene enrichment. Adv Exp Med Biol.

[b21] Gwinn ML, Stellwagen AE, Craig NL, Tomb JF, Smith HO (1997). In vitro Tn7 mutagenesis of *Haemophilus influenzae* Rd and characterization of the role of *atpA* in transformation. J Bacteriol.

[b22] Herriott RM, Meyer EM, Vogt M (1970). Defined nongrowth media for stage II development of competence in *Haemophilus influenzae*. J Bacteriol.

[b23] Johnsborg O, Havarstein LS (2009). Regulation of natural genetic transformation and acquisition of transforming DNA in *Streptococcus pneumoniae*. FEMS Microbiol Rev.

[b24] Kristensen BM, Sinha S, Boyce JD, Bojesen AM, Mell JC, Redfield RJ (2012). Natural transformation of *Gallibacterium anatis*. Appl Environ Microbiol.

[b25] Livermore DM (2012). Fourteen years in resistance. Int J Antimicrob Agents.

[b26] Lo Scrudato M, Blokesch M (2012). The regulatory network of natural competence and transformation of *Vibrio cholerae*. PLoS Genet.

[b27] Ma C, Redfield RJ (2000). Point mutations in a peptidoglycan biosynthesis gene cause competence induction in *Haemophilus influenzae*. J Bacteriol.

[b28] Macfadyen LP (2000). Regulation of competence development in *Haemophilus influenzae*. J Theor Biol.

[b29] Macfadyen LP, Chen D, Vo HC, Liao D, Sinotte R, Redfield RJ (2001). Competence development by *Haemophilus influenzae* is regulated by the availability of nucleic acid precursors. Mol Microbiol.

[b30] Maughan H, Sinha S, Wilson L, Redfield RJ (2008). Pasteurellaceae: Biology, Genomics and Molecular Aspects.

[b31] Meibom KL, Blokesch M, Dolganov NA, Wu CY, Schoolnik GK (2005). Chitin induces natural competence in *Vibrio cholerae*. Science.

[b32] Miller JH (1992). A Short Course in Bacterial Genetics: A Laboratory Manual and Handbook for *Escherichia coli* and Related Bacteria.

[b33] Mironov AA, Koonin EV, Roytberg MA, Gelfand MS (1999). Computer analysis of transcription regulatory patterns in completely sequenced bacterial genomes. Nucleic Acids Res.

[b34] Palchevskiy V, Finkel SE (2006). *Escherichia coli* competence gene homologs are essential for competitive fitness and the use of DNA as a nutrient. J Bacteriol.

[b35] Pifer ML, Smith HO (1985). Processing of donor DNA during *Haemophilus influenzae* transformation: analysis using a model plasmid system. Proc Natl Acad Sci USA.

[b36] Poje G, Redfield RJ (2003). Transformation of *Haemophilus influenzae*. Methods Mol Med.

[b37] Pollack-Berti A, Wollenberg MS, Ruby EG (2010). Natural transformation of *Vibrio fischeri* requires *tfoX* and *tfoY*. Environ Microbiol.

[b38] Ravcheev DA, Gel'fand MS, Mironov AA, Rakhmaninova AB (2002). Purine regulon of gamma-proteobacteria: a detailed description. Genetika.

[b39] Redfield RJ (1991). *sxy*-1, a *Haemophilus influenzae* mutation causing greatly enhanced spontaneous competence. J Bacteriol.

[b40] Redfield RJ (1993). Genes for breakfast: the have-your-cake-and-eat-it-too of bacterial transformation. J Hered.

[b41] Redfield RJ, Cameron AD, Qian Q, Hinds J, Ali TR, Kroll JS, Langford PR (2005). A novel CRP-dependent regulon controls expression of competence genes in *Haemophilus influenzae*. J Mol Biol.

[b42] Redfield RJ, Findlay WA, Bosse J, Kroll JS, Cameron AD, Nash JH (2006). Evolution of competence and DNA uptake specificity in the Pasteurellaceae. BMC Evol Biol.

[b43] Schneider TD, Stormo GD, Gold L, Ehrenfeucht A (1986). Information content of binding sites on nucleotide sequences. J Mol Biol.

[b44] Seitz P, Blokesch M (2012). Cues and regulatory pathways involved in natural competence and transformation in pathogenic and environmental Gram-negative bacteria. FEMS Microbiol Rev.

[b46] Sinha S, Redfield RJ (2012). Natural DNA uptake by *Escherichia coli*. PLoS ONE.

[b1001] Sinha S, Cameron ADS, Redfield RJ (2009). Sxy induces a CRP-S regulon in *Escherichia coli*. J Bacteriol.

[b45] Sinha S, Mell JC, Redfield RJ (2012). Seventeen Sxy-dependent cyclic AMP receptor protein site-regulated genes are needed for natural transformation in *Haemophilus influenzae*. J Bacteriol.

[b47] Solomon JM, Grossman AD (1996). Who's competent and when: regulation of natural genetic competence in bacteria. Trends Genet.

[b48] Stewart GJ, Carlson CA (1986). The biology of natural transformation. Annu Rev Microbiol.

[b49] Suckow G, Seitz P, Blokesch M (2011). Quorum sensing contributes to natural transformation of *Vibrio cholerae* in a species-specific manner. J Bacteriol.

[b50] Taylor LA, Rose RE (1988). A correction in the nucleotide sequence of the Tn903 kanamycin resistance determinant in pUC4K. Nucleic Acids Res.

[b51] Taylor RG, Walker DC, McInnes RR (1993). *E. coli* host strains significantly affect the quality of small scale plasmid DNA preparations used for sequencing. Nucleic Acids Res.

[b52] Tracy E, Ye F, Baker BD, Munson RS (2008). Construction of non-polar mutants in *Haemophilus influenzae* using FLP recombinase technology. BMC Mol Biol.

[b53] Warming S, Costantino N, Court DL, Jenkins NA, Copeland NG (2005). Simple and highly efficient BAC recombineering using *galK* selection. Nucleic Acids Res.

[b54] Wetmore PW, Thiel JF, Herman YF, Harr JR (1963). Comparison of selected *Actinobacillus* species with a hemolytic variety of *Actinobacillus* from irradiated swine. J Infect Dis.

[b56] Yamamoto S, Morita M, Izumiya H, Watanabe H (2010). Chitin disaccharide (GlcNAc)2 induces natural competence in *Vibrio cholerae* through transcriptional and translational activation of a positive regulatory gene *tfoXVC*. Gene.

[b55] Yamamoto S, Izumiya H, Mitobe J, Morita M, Arakawa E, Ohnishi M, Watanabe H (2011). Identification of a chitin-induced small RNA that regulates translation of the *tfoX* gene, encoding a positive regulator of natural competence in *Vibrio cholerae*. J Bacteriol.

[b57] Zhang Y, Morar M, Ealick SE (2008). Structural biology of the purine biosynthetic pathway. Cell Mol Life Sci.

