# Supplementary material for: The availability of purine nucleotides regulates natural competence by controlling translation of the competence activator Sxy
Source: Mol Microbiol. 2013 May 13;88(6):1106–19. doi: 10.1111/mmi.12245 (PMC3739930; doi:10.1111/mmi.12245)

Table S1: Primers used in this study.

Name	Sequence	Target
purRF	CCACCACAGAACCAGCAGGTA	amplify *purR* and flank
purRR	GCTTTCCAACGGGCAAA	
purRinv_F	GGGGTACCCGTTTAGGCAGACAGGCAAT	for inverse PCR on pG*purR*
purRinvR	AAAGATGTGGCAAAAATGGCGAATTCCG	
purHF	CGGATTGGTGTGTTGCTTGTA	amplify *purH* and flank
purHR	AACTCTGTCTTAAAGCATGTGACG	
purHinv_F	CGGAATTCCGGCGGTTTATTAGTACAAGAT	for inverse PCR on pG*purH*
purHinvR	CCCAAGCTTCGCCATGTACTTTGGGATGTAA	
purFF	GGCGAAGTTAGGCATTATTTT	amplify *purF* and flank
purFR	GGAACAGGACTTATCGGCAA	
ΔpurFF	GAGTTCAAGTTTTTTAACTCCAACGCTTAATCAATAAGGAACAC AAAATGATTCCGGGGATCCGTCGACC	mutagenic primers for recombineering
ΔpurFR	TGATTTTTATCCCTCTAAATGCCTAGCATTTATTGTTCATTGTA AATTTCTGTAGGCTGGAGCTGCTTCG	
hisFF	CTGTCCGTTATTTGATGGTAT	amplify *hisF* and flank
hisFR	GCTGCCTACAGTTTTGTT	
ΔhisFF	GGTCGAGCTTTATTAGAAGGAAAATTTACGCTTTCGGAGGCAAT CAAATGATTCCGGGGATCCGTCGACC	mutagenic primers for recombineering
ΔhisFR	TTTTTGCCAGTCTATTTTAGTAATATTCATTATTCACTCCGAAT CTCGATTGTAGGCTGGAGCTGCTTCG	
hfqF	GCGATGAAAAGTGCGGTAGA	amplify *hfQ* and flank
hfqR	GTGCGAGTAAACAAAGGCAGAA	

ΔhfQF	CGGGATCCCCATTTGATTCACGGTGTTTT	mutagenic primers for recombineering
ΔhfQR	CGGGATCCCTGCTCGTTCAGTTTCCCAT	
AssxyF	CCAGCTTCAACACCTGCTTT	amplify *sxy* and flank from *A. suis*
AssxyR	TTATCGGCGTTATATTCGGC	
ΔAssxyF	CATAATGAATATAAAGTTACAAAAGTAAATCATCAAAAGGAGCT CGTATGATTCCGGGGATCCGTCGACC	mutagenic primers for recombineering
ΔAssxyR	CCAAATGGTCGGTTTTTTTATGTCTGACTTTATTTTACGCACAG CATGAATGTAGGCTGGAGCTGCTTCG	

Fig. S1: The addition of purines does not affect cell viability. Viable counts on plain medium after 100 mins in MIV in the presence (grey bars) or absence (black bars) of purine nucleotide addition (top graph: 1 mM AMP, bottom graph: increasing AMP concentrations as stated). . Each bar or point represents the mean of three biological replicates ± standard deviations. Circles: WT (wildtype), squares: Δ*purH*, triangles: *sxy*-1.

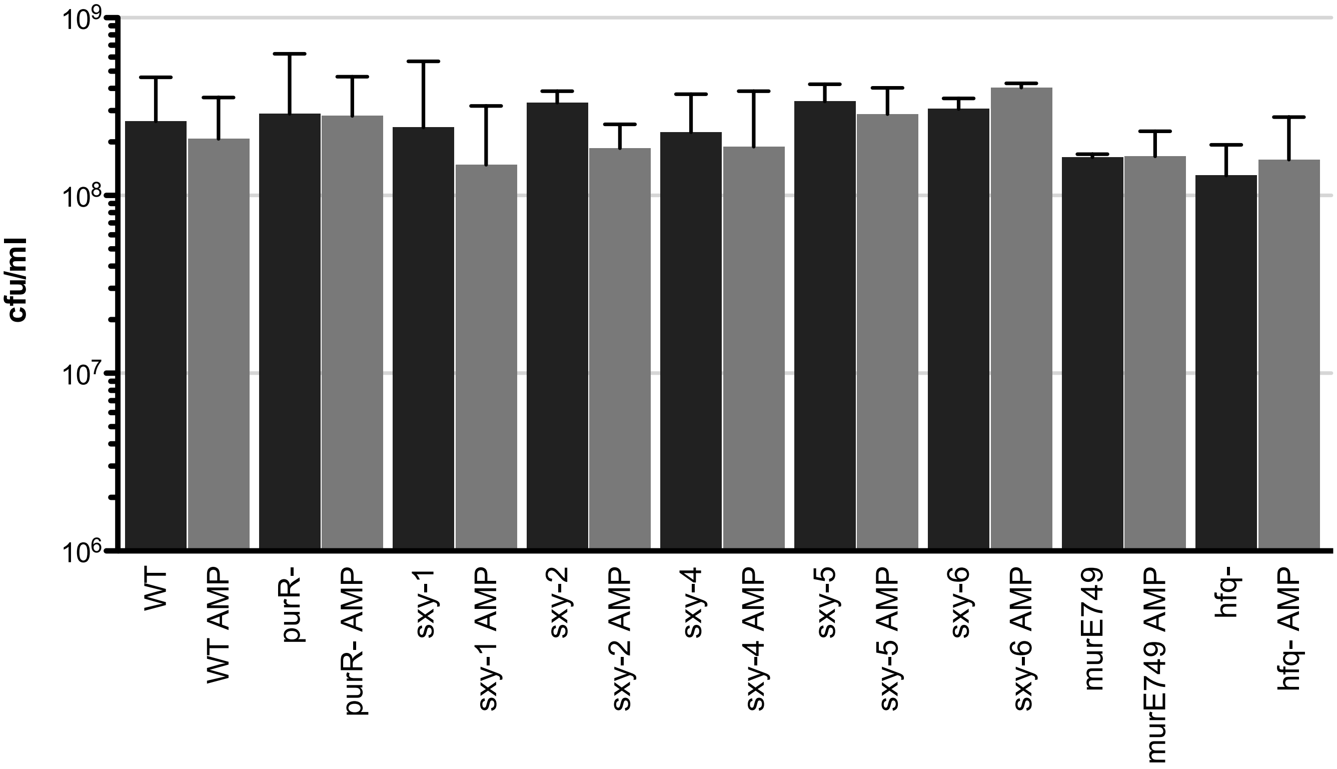

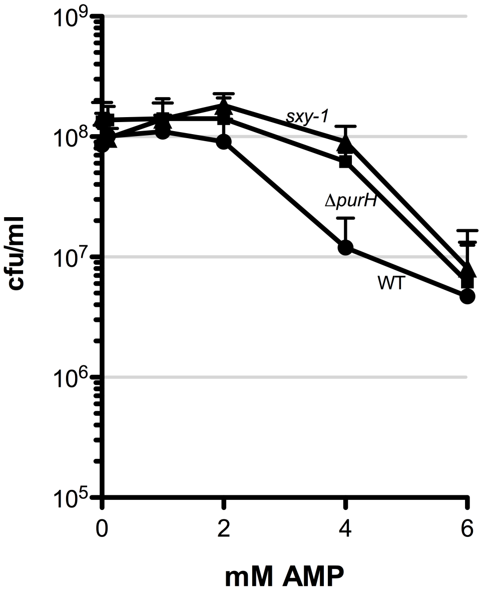

Fig. S2: Growth of the *purR*- strain in sBHI broth. The OD_{600} of the wildtype (black circles) and *purR*- (grey squares) strains was sampled every hour or 30 min (top) and the number of viable cells determined by plating (bottom). Error bars represent standard deviation from the mean of two replicates.

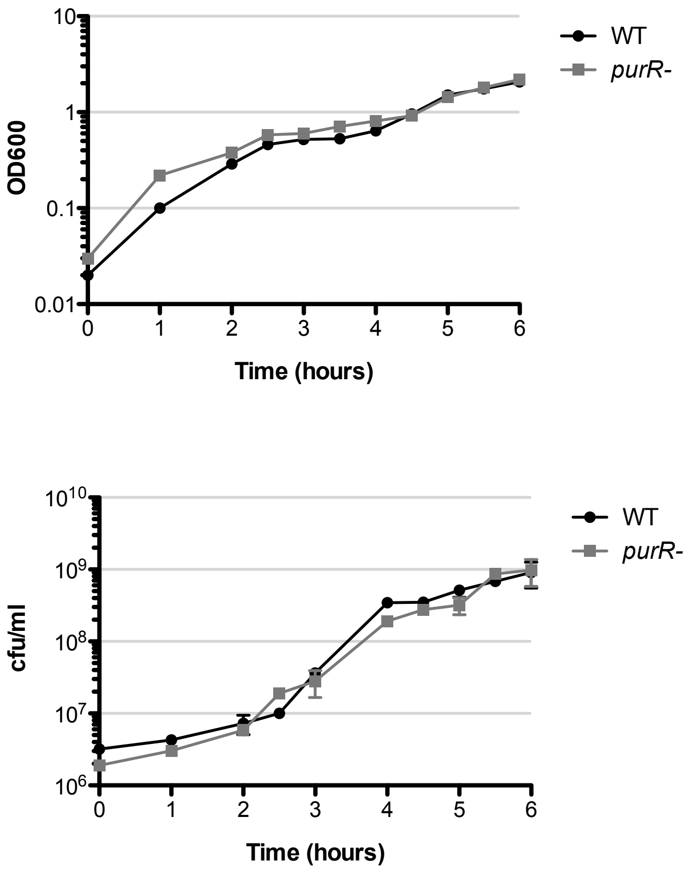

Supplement: Supplementary file 1 [file mmi0088-1106-SD1.pdf]
